# Macrophage Regnase-1 Deletion Deteriorates Liver Ischemia/Reperfusion Injury Through Regulation of Macrophage Polarization

**DOI:** 10.3389/fphys.2020.582347

**Published:** 2020-10-29

**Authors:** Ai Xiaoming, Jia Wenbo, Wang Jinyi, Wu Bin, Hu Chunyang, Chen Qi, Kong Lianbao

**Affiliations:** ^1^Liver Transplantation Center, The First Affiliated Hospital of Nanjing Medical University, Nanjing, China; ^2^Department of General Surgery, BenQ Medical Center, The Affiliated BenQ Hospital of Nanjing Medical University, Nanjing, China

**Keywords:** regnase-1 (MCPIP), liver, ischemia/reperfusion injury, macrophage, polarization

## Abstract

**Background:**

Regnase-1 (MCPIP) has been identified as an anti-inflammatory agent, but little is known about its influence on liver ischemia/reperfusion (I/R) injury. Macrophages can evolve biphasic responses and differentiate into remarkable polarizations, contributing greatly to the uncontrolled inflammatory cascades during liver I/R injury. Therefore, the aim of this study was to explore whether regnase-1 participated in liver I/R via manipulating macrophage polarization.

**Materials and methods:**

C57BL/6 mice were randomly divided into five groups: Sham, I/R, Clodronate, Clo + BMDM, and Clo + LV MCPIP BMDM. A liver I/R model was established, and histopathological and immunostaining examinations were performed for the liver specimens; double immunofluorescence staining was used to localize MCPIP in the liver. Primary hepatocytes were isolated to simulate a hypoxia and reoxygenation (H/R) model *in vitro*. Bone marrow-derived macrophages (BMDM) were extracted and subjected to lentiviral transduction to knockdown MCPIP expression. BMDM with or without MCPIP deletion were exposed to H/R supernatants, and the polarized states were measured by flow cytometry. RT-PCR analysis and Western blot were also conducted.

**Results:**

Compared to those in the Sham group, liver functions and Suzuki’s scores were deteriorated in the I/R group, which were reversed in the Clodronate group. The increased expression of regnase-1 in the I/R group diminished with pretreatment of clodronate liposomes. Subsequent double immunofluorescence staining established the localization of regnase-1 in macrophages in the liver. The insulted lesions in the Clodronate group became progressively aggravated with adoptive transfer of BMDM in the Clo + BMDM group, and they were further exacerbated with the transfusion of BMDM with MCPIP knockdown in the Clo + LV MCPIP BMDM group. Gene expressions of M1 and M2 markers were detected by RT-PCR, suggesting that MCPIP knockdown tended to favor the M1 transformation. Subsequently, *ex vivo* flow cytometrical detection showed that, upon stimulation by H/R supernatants, LV-MCPIP BMDM posed a higher ratio of M1/M2 than BMDM. Finally, we found that MCPIP participated in macrophage M1/M2 polarization through the NF-κB, C/EBPβ, and PPARγ signaling pathways during liver I/R.

**Conclusion:**

Our study confirms that regnase-1 plays a critical role in liver I/R via regulation of macrophage polarization and, thus, might offer a potential therapeutic target.

## Introduction

The pathogenesis of ischemia/reperfusion (I/R) involves an initial deprivation of blood supply and a subsequent restoration of perfusion, featuring an extensive inflammatory response and consequently exacerbated tissue injury (Qiu et al., 2017; Wan et al., 2020). Hepatic I/R injury is a very common condition during major liver surgeries, but the complex mechanisms remain enigmatic. In fact, it is an acute and dynamic process, throughout which the innate and adaptive immune systems experience unprecedented irritations, persistently driving the ensuing amplified cascades downstream. Specifically, hepatic I/R poses microcirculation disturbance at first, triggers reactive oxygen species (ROS) production subsequently, and then activates the innate immune responses (Li Z. et al., 2018). Upon activation, the very innate immunocytes, particularly as macrophages (mø) and neutrophils, release large amounts of inflammatory cytokines, deteriorating the hepatic dysfunction and eventually causing cell death (Yu et al., 2016; Li Z. et al., 2018). Kupffer cells (KCs), the liver-resident macrophages, which are representative of the largest fixed population of macrophages for the host, are dedicated to the maintenance of liver homeostasis. They are essentially engaged in the innate immune responses during hepatic I/R.

However, the detailed role of macrophages has long been conflicting during the course of liver I/R because they are volunteered to elicit this sterile inflammation but can evolve biphasic responses and differentiate into remarkable polarizations due to their heterogeneity and plasticity (Kapoor et al., 2015). Generally speaking, two polarizations of activated macrophages are defined, namely M1 and M2, which are endowed with distinct phenotypes and functions (Hu et al., 2019). M1, as the “killer” type, are occupied in the clearance of pathogens and promotion of inflammation, and M2, the “heal” type, function in tissue repair and resolution of inflammation. M1 can be sensitized by mediators, including lipopolysaccharides (LPS), IL-1β, and IFN-γ, and are characterized by their production of proinflammatory cytokines, such as TNF-a, IFN-γ, IL-6, IL-12, ROS, and so on, whereas, M2 are induced by IL-4 and IL-13 and devoted to the formation of anti-inflammatory markers such as IL-10, resirtin-like-a (Fizz1), Arginase 1 (Arg1), and Chitinase 3-like (Ym1) (Kapoor et al., 2015). Macrophage polarization has been widely documented to occur under a variety of circumstances, either pathologically or physiologically (Kang and Lee, 2016). Mounting evidence supports the critical role of macrophage polarization in hepatic I/R, declaring that an imbalance of M1/M2 polarization contributes to the uncontrolled inflammatory cascades (Kapoor et al., 2015).

Regnase-1, alias monocyte chemoattractant protein-1 induced protein (MCPIP1), or Zc3h12a, is known by researchers for its immunosuppressive properties. It was originally discovered as a novel CCCH-type zinc-finger protein in human peripheral blood monocytes, caused by monocyte chemoattractant protein-1 (MCP-1) coupling with its receptor CCR2 (Niu et al., 2013). It is well known nowadays that this protein is encoded by the ZC3H12A gene, which can be excited by a number of inflammatory cytokines, like MCP-1, IL-1β, TNF-α, and bacterial LPS, upon nuclear factor kappa-B (NF-κB) activation (Niu et al., 2013; Bugara et al., 2017). It is present in various types of cells but extremely enriched in immune cells, such as macrophages and monocytes, and is mostly localized in the cytoplasm and endoplasmic reticulum (ER) to exert its bioactivity (Fu and Blackshear, 2017).

Three main peculiar structures harbored in regnase-1 intrinsically confer multiple special properties and functions. Thereby, it is a versatile protein and can function as a transcription factor, deubiquitinase, endoribonuclease (RNase), and RNA binding protein (RBP) in certain scenarios (Behrens et al., 2018). Accordingly, regnase-1 can not only recognize stem-loop structures in 3′UTRs of target mRNAs to accelerate their destabilization (Cui et al., 2017), but it also directly degrades a large set of proinflammatory cytokines, including IL-6, IL-12p40, IL-1β, and several microRNAs in innate immunocytes (Takaishi et al., 2018). Moreover, regnase-1 acts to suppress the NF-κB signaling pathway, a key player for inflammatory response, thus reputed as a negative regulator of inflammation in a cross-regulatory fashion (Bugara et al., 2017; Ligeza et al., 2017; von Gamm et al., 2019). Regnase-1-deficient (Zc3h12a−/−) mice, which are predisposed to severe autoinflammatory diseases that end in lethality, have a particular battery of elevated proinflammatory cytokines infiltrating their organs, especially the liver and lung, including IL-6 and IL-12p40, due to failure in destructing the encoding mRNAs (Jeltsch et al., 2014; Mino et al., 2019).

Hence, researchers have come to realize that regnase-1 is rather essential for immune homeostasis and have paid increasing attention to its therapeutic potential for immune modulation. However, whether regnase-1 affects the process of hepatic I/R is still unknown, and knowledge about its regulatory role in macrophage polarization retains an ill-defined issue. Although studies on liver I/R have made enormous achievements, regulation of macrophage polarization during I/R invariably deserves to be appreciated, and reciprocal relationship between regnase-1 and the balance of M1/M2 polarization warrants exploration. In such a context, we attempted to elucidate the immunomodulatory effect of regnase-1 on liver I/R in this study and to further excavate the role of regnase-1 in governing macrophage M1/M2 phenotypic transformation thereof. This study was designed to lend new insights into the sophisticated mechanisms of liver I/R and aimed to provide potential therapeutic strategies to control the relevant sterile inflammation.

## Materials and Methods

### Animals and Experimental Protocol

Male, wild-type C57BL/6 mice aged 6–8 weeks were purchased from Qinglongshan Animal Breeding Field in Jiangning District, Nanjing City. All mice were housed in specific pathogen-free facilities and fed with free access to standard chow and tap water. Animal breeding and treatment in our study were in compliance with the guidelines of the Chinese Council on Animal Care, and the experimental protocols were censored and approved by the Animal Care and Use Committee of Nanjing Medical University. All the animals were kept fasted for 12 h prior to surgery and allowed to drink *ad libitum*.

The mice were randomly allocated into five groups in our studies (*n* = 6) as follows:

(i) The sham-operated group (Sham), in which only a ventral laparotomy was performed.
(ii) The I/R group (I/R) undergoing an operation to create a hepatic I/R model.
(iii) The clodronate liposomes group (Clodronate). Herein, a dose of 200 μl clodronate liposomes (from Vrije Universiteit Amsterdam) was injected into each mouse via the tail vein 24 h prior to the establishment of a hepatic I/R model to deplete macrophages *in vivo*.
(iv) The clodronate liposomes + macrophage group (Clo + BMDM). In this group, apart from the application of clodronate liposomes 24 h preoperatively, approximately 1 × 10^6^ macrophages suspended in 100 μl of PBS were infused back intravenously to each one, just 1 h before the onset of I/R injury to reconstitute macrophages *in vivo*.
(v) The clodronate liposomes + LV-MCPIP macrophage group (Clo + LV-MCPIP) underwent the same maneuvers as the Clo + mø group, but for infusion of the genetically modified macrophages with MCPIP insufficiency by lentiviral (LV) transduction instead.

### Liver I/R Model

A mouse model of segmental (70%) hepatic warm I/R was established as depicted previously (Huang et al., 2015; Hu et al., 2019). In brief, under anesthesia with isoflurane inhalation, male C57BL/6 mice underwent a midline laparotomy adopting aseptic techniques. After careful exposure of the portal triad, vessel branches supplying the left and middle liver lobes were interrupted with an atraumatic clip for 90 min with blood supplies to the right and caudate lobes left intact. Thereafter, the clip was removed, and reperfusion ensued. Body temperature was monitored and maintained at 37°C during the entire surgery. The sham-operated animals underwent the same procedure without vascular occlusion. At 6 h following reperfusion, the mice were sacrificed for blood collection and liver tissue specimens.

### Isolation and Culture of Primary Hepatocytes

Normal, wild-type C57BL/6 mice were utilized to isolate primary hepatocytes as delineated with minor modifications (Czaya et al., 2017). In short, in a completely sterile scenario, the portal vein was cannulated with a shielded i.v. catheter, and the liver was perfused with 1 × Hanks’ Balanced Salt Solution (HBSS) (Invitrogen Life Technologies) to flush red cells. The infrarenal IVC was transected to permit suitable drainage of blood and perfusion media. Following 20 ml of 1 × HBSS, 20 ml of liver digest media containing collagenase type IV were continued. After that, the liver was excised and transferred to a sterile tissue culture dish, wherein it was minced, and undigested tissue particles and cell debris were discarded through a 70-μm nylon mesh cell strainer. After centrifugation at 50 × *g* at 4°C for 3 min thrice, the primary hepatocytes were obtained and incubated in Dulbecco’s modified Eagle medium (DMEM) supplemented with 10% fetal bovine serum (FBS) on collagen-coated six-well plates. Four hours after cell adherence, the media were replaced with fresh DMEM supplemented with 10% FBS, followed by media change every 24 h for the maintenance of primary hepatocytes.

### Primary Hepatocyte Hypoxia and Reoxygenation (H/R)

Freshly acquired primary hepatocytes were prepared to simulate an H/R model *in vitro* as described (Tan et al., 2018; Yang et al., 2019). The hepatocytes were seeded at a density of 1 × 10^6^ cells/ml and cultured in a standard incubator (95% air and 5% CO_2_, 37°C) with 21% normoxic O_2_. To expose them to H/R, the cells were placed in serum- and glucose-free deoxygenated DMEM medium and challenged to a hypoxic environment (1% O_2_, 5% CO_2_, and 94% N_2_) in a specially designed hypoxic incubator under constant temperature and humidity for 1.5 h. Subsequently, the plates were retrieved, substituted once again with fresh DMEM supplemented with 10% FBS, and returned to the standard incubator under a normoxic state for another 6 h to achieve reoxygenation. The cells and supernatants were then collected for further experiments.

### Depletion of Macrophages

Because clodronate liposomes have been widely accepted as an effective and pleiotropic method to deplete macrophages *in vivo* in light of the literature (Kozicky and Sly, 2019), we decided to take advantage of this drug to constitute a mouse model of macrophage depletion in C57BL/6 mice. A single dose of 200 μl clodronate liposomes was injected into each mouse via the tail vein 24 h prior to the surgery to eliminate of all types of macrophages.

### Isolation and Culture of Bone Marrow-Derived Macrophages (BMDM)

Wild-type C57BL/6 mice were exploited to isolate BMDM from the bone marrow as previously described (Ying et al., 2013; Kozicky and Sly, 2019). Briefly, the mouse was euthanized and sterilized with 70% ethanol. The skin and fur were cleared off each of the hind legs, the muscles were trimmed, and the tibias and femurs exposed. A 26-gage needle was used to flush the bone marrow into a 50 mL conical tube. After centrifugation at 300 × *g* at 4°C for 5 min, BMDM were initially acquired and cultured in DMEM supplemented with 10% FBS. Importantly, recombinant granulocyte-macrophage colony-stimulating factor (GM-CSF) with a dose of 20 ng/ml were necessary to induce differentiation of BMDM into normal macrophages (M0) over time. On day 7 after initial culture, BMDM were observed under an inverted microscope and then ready for use.

### Reconstitution of Macrophages

Some of the C57BL/6 mice subjected to clodronate liposome injection were taken to reconstitute macrophages *in vivo* via adoptive transfer of BMDM (M0) as delineated previously (Kozicky and Sly, 2019). To this end, the prepared macrophages were resuspended in PBS with a concentration of 1 × 10^7^ cells/ml; 100 μl of the macrophage solution (1 × 10^6^ cells) filled a 1 ml syringe with a 26-gage needle and was infused back into the mouse via the tail vein at 1 h preoperatively. For some others, the genetically modified macrophages with MCPIP knockdown were employed instead of the macrophages themselves.

### Flow Cytometry

We utilized flow cytometry to assess the classical macrophage expression of F4/80-PerCP, CD11b-FITC, M1 marker CD86-PE, and M2 marker CD206-APC, according to the manufacturer’s instructions. The prepared BMDM (M0) (5 × 10^5^ cells) were resuspended in 100 μl cell staining buffer into 12 mm × 75 mm plastic tubes. To stain cell surface antibodies, 1 μg Anti-F4/80, 1 μg Anti-CD11b, and 1 μg Anti-CD86 were added to the macrophage solutions, respectively, and incubated on ice for 20 min in the dark. These initially stained solutions were washed twice by 2 ml cell staining buffer and centrifuged at 350 *g* for 5 min. The cells were fixed in 0.5 ml fixation buffer in the dark for 20 min, followed by centrifugation and then resuspended in 2 ml intracellular staining perm wash buffer. Then, 1 μg anti-CD206-APC were added to these cells and incubated on ice for 20 min in the dark to gain intracellular staining. At last, the intracellularly labeled cells were washed again with intracellular staining perm wash buffer and resuspended in 0.5 ml cell staining buffer, which were, at this point, tested on the flow cytometer and analyzed by Flow Jo v10.0.7 software.

### Induction of Macrophage M1/M2 Polarization

A well-established approach was adapted to induce macrophage polarization (Ying et al., 2013). Briefly speaking, the aforementioned BMDM were seeded at a density of 1 × 10^6^ cells/ml and induced into macrophages (M0). On day 7, the cells were changed with fresh DMEM supplemented with 10% FBS. At the same time, 100 ng/ml LPS was added to the culture for the M1 polarizing regimen while 10 ng/ml IL-4 was applied for the M2 regimen. The cells kept on differentiating for 24 h and then collected for further evaluations.

### Hepatocellular Function Assay

Blood samples were collected 6 h following reperfusion, and sera were separated from the whole blood after centrifugation. Levels of serum alanine aminotransferase (ALT) and aspartate transaminase (AST) were measured by an automatic biochemical meter (Beckman CX7; Beckman Coulter, CA, United States), which served as indicators of hepatocellular injury.

### Lentiviral Transduction of Macrophages

Lentiviral infection into macrophages was performed to knock down MCPIP expression, following the manufacturer’s protocol. LV vectors expressing short hairpin RNAs were purchased from Jiangsu KeyGEN BioTECH Corp., Ltd. In short, LV-Enhance (50×) was first diluted in DMEM supplemented with 10% FBS to get LV-Enhance (1×) as appropriate. BMDM (M0) were seeded at a concentration of 1 × 10^6^ cells/ml. After PBS wash, the cells were cultured in LV-Enhance (1×) containing media and, in the meantime, were mixed with LV vectors with 30–50 multiplicities of infection (MOI). To attain genetic transduction, the infected macrophages were kept growing for 3 days, but if cell viability became compromised, the culture was substituted by fresh DMEM supplemented with 10% FBS in time. After being checked under an inverted microscope, the genetically modified macrophages were ready for other examinations.

### Quantitative Real-time Polymerase Chain Reaction Analysis (RT-PCR)

Quantification of target mRNA expression was determined by RT-PCR analysis, according to the manufacturer’s recommendations. Total RNA extracted by TRIzol reagent (Invitrogen, Carlsbad, CA, United States) were reverse transcribed to synthesize cDNAs using a SuperScript III System (Invitrogen, Carlsbad, CA, United States). The PCR reaction mixture was prepared with the SYBR Green Master Mix (Roche, Indianapolis, IN, United States), and amplification conditions were accomplished at an initial denaturation phase at 94°C for 5 min, followed by 45 cycles, then denaturation at 94°C for 45 s, and then annealing at 58°C for 60 s, and eventually, extension at 72°C for 60 s. Taking advantage of specific primers, relative levels of target gene expression were quantified by the comparative cycle threshold (CT) method (ΔΔCT) with normalization against ß-actin mRNA.

### Western Blotting Analysis

Western blotting analysis was completed to measure protein expression levels, as per the instruction manual. Generally, tissue or cellular proteins were homogenized and extracted by RIPA lysis buffer (Wako, Osaka, Japan), and protein concentrations were determined using a BCA protein quantitative kit (Abcam, United States). The extracted proteins were submitted to electrophoresis in 12% sodium dodecyl sulfate-polyacrylamide gel electrophoresis (SDS-PAGE) for separation and transferred onto polyvinylidene difluoride (PVDF) membranes. The membranes were incubated overnight with primary indicated antibodies, accompanied by incubation in horseradish peroxidase conjugated secondary antibodies (Abcam, MA, United States). The developed blots were visualized by enhanced chemiluminescence on the Kodak Image Station (Carestream Health, Rochester, NY, United States) while target band intensity was normalized to that of GAPDH.

### Histopathological and Immunostaining Examination

Liver specimens from the ischemic lesions were fixed in 10% buffered formaldehyde, dehydrated with ethanol in xylene, embedded in paraffin, and sectioned at 5 μm. Then, the serial sections (5 μm) were mounted on glass slides and stained using hematoxylin-eosin (H&E). For histological examination, the slides were inspected under light microscopy by a histopathologist blinded to the design. Suzuki’s criteria were quoted to assess the severity of inflammation and tissue damage (Suzuki et al., 1993). A score ranging from 0–4 was assigned to each slide in accordance with the criteria, which indicates that the higher the score, the more severe the injury.

Immunohistochemical examination was carried out to visualize the presence of KCs in the form of F4/80, a macrophage marker. The liver tissues were embedded in optimum cooling temperature compound (Tissue-Tek, Sakura Finetek, Torrance, CA, United States), frozen promptly in liquid nitrogen at −80°C and sliced at 6 μm. After being deparaffinized and rehydrated in graded concentrations of ethanol, the serial slices were mounted on glass slides. The slides were submitted to a battery of fixation, permeabilization, antigen retrieval, and blockage and incubated in diluted antirabbit F4/80 as the primary antibody, followed by incubation in others as the second antibodies, and then counterstained in 3,3′-diaminobenzidine (DAB; Nichirei Inc., Tokyo, Japan) and hematoxylin for visualization. Finally, the slides were photographed and semiquantified by an inverted microscope, invariably in a blinded fashion.

### Double Immunofluorescence Staining

To gain insight into the location and expression level of regnase-1 (MCPIP) in KCs in the liver under different milieu, double immunofluorescence was performed with a combination of diluted antirabbit F4/80 and monoclonal antibody against MCPIP. Fresh frozen liver tissue sections on coverslips from all groups were sequentially reacted with F4/80 and anti-MCPIP for staining as the primary antibody overnight, followed by reaction with horseradish peroxidase-conjugated secondary antibody (Histofine simple stain MAX PO; Nichirei Inc., Tokyo, Japan) as the second antibody. The nuclei were finally counterstained by 4′,6-diamidino-2-phenylindole (DAPI) for 3 min for cell localization. Fluorescence images were captured under an LSM PASCAL confocal laser scanning microscope (Carl Zeiss, Oberkochen, Germany), with excitation spectral laser lines at 405 and 594 nm.

### Statistical Analysis

All results were representative of three independent experiments. Data were presented as mean ± SD. Differences between two groups were examined by Student’s *t*-test, and ANOVA was used for comparison of multiple groups. *P* values less than 0.05 were deemed statistically significant.

## Results

### To Validate the Specific Role of Macrophages in Hepatic I/R Injury

**FIGURE 1:**
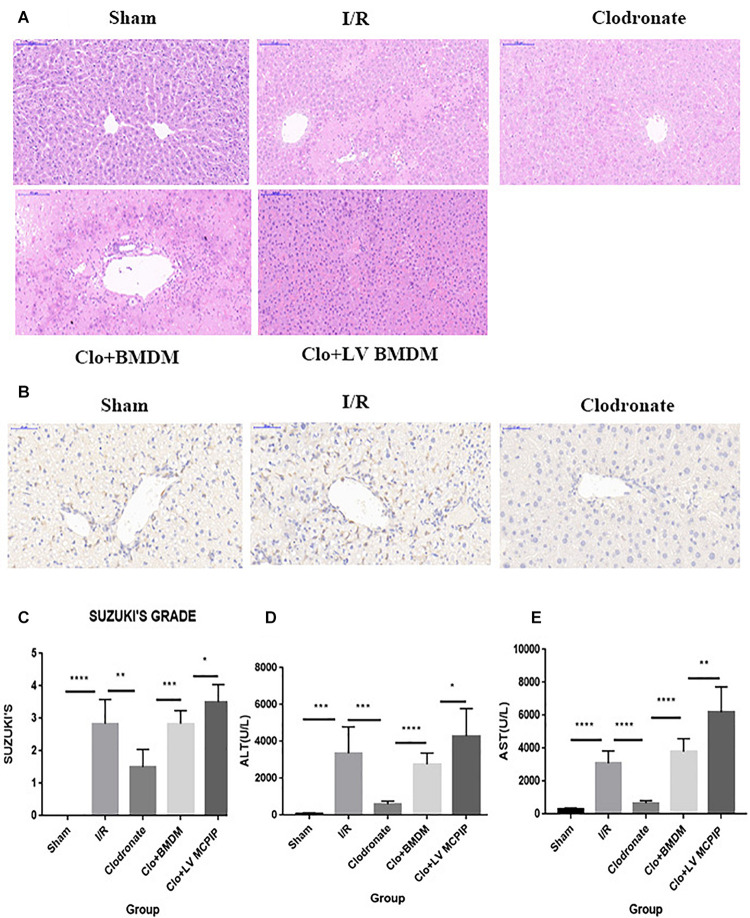
MCPIP knockdown aggravated liver IR injury in C57BL/6 mice. **(A)** Representative histological staining using hematoxylin-eosin (H&E) was demonstrated. Regarding congestion, vacuolization, and necrosis, livers in the I/R group showed severe impairments compared to those in the Sham group, but this severity was reversed in the Clodronate group. Impairments in the Clo + BMDM group turned aggravated again, which became worse in the Clo + LV-MCPIP DMBM group. **(B)** Immunohistochemical examination was performed to visualize the presence of Kupffer cells as F4/80 positive. The markedly increased F4/80 positive cells in the I/R group were observably absent in the Clodronate group. **(C)** Suzuki’s histological score was quantified. **(D,E)** Hepatocellular function was assayed by serum ALT and AST levels. *P < 0.05, **P < 0.01, ***P < 0.005, and ****P < 0.001.

Male C57BL/6 mice were chosen to establish the hepatic I/R model, and the degree of liver damage was evaluated by the hepatocellular function assays and Suzuki’s scores (Figure 1). First of all, serum ALT and AST levels were detected to be profoundly increased 6 h after reperfusion in the I/R group compared with corresponding levels in the Sham group (Figures 1D,E). Second, histologically, affected tissues were observed microscopically, and a score was awarded to each slide in line with Suzuki’s criteria (Suzuki et al., 1993). As a result, livers from the I/R group showed characteristically more disordered hepatic architecture, pronounced lobular edema, and sinusoidal congestion, heavy centrilobular ballooning as well as extensive hepatocellular necrosis than those in the Sham group, and Suzuki’s scores were obviously higher in the I/R group (Figures 1A,C). Thereupon we were convinced that the hepatic I/R model complicated by massive disturbed areas and severe tissue damage was successfully established in our study.

Given the well-established involvement of macrophages in hepatic I/R, we wondered whether the injurious effect in I/R was macrophage specific. A dose of clodronate liposomes were administered intravenously to mice to deplete all types of macrophages *in vivo* 24 h prior to surgery. The depletion efficiency was determined by immunohistochemical examination. As shown in Figure 1B, macrophages were typically labeled with F4/80. As more than 90% of F4/80-positive cells were observably absent, the efficacy was considered highly appreciable.

Intriguingly, macrophage exhaustion in the Clodronate group led to recovered affected lesions after reperfusion compared with those in the I/R group (Figures 1A,C). To further test the specific role of macrophages, we transfused BMDM (M0) back into mice pretreated with clodronate liposomes to reconstitute macrophages 1 h before the onset of I/R. The recovery in the Clodronate group once again worsened upon macrophage reconstitution in the Clo + mø group. In parallel, serum levels of ALT and AST were detected, which displayed similar patterns with the histopathological results (Figures 1D,E).

To summarize, macrophages play a critical role in hepatic I/R injury, whereas macrophage elimination is responsible for the mitigated insults, suggestive of the macrophage-specific effect, in accordance with the literature.

### To Designate the Varied Expression of Regnase-1 (MCPIP) During I/R

**FIGURE 2:**
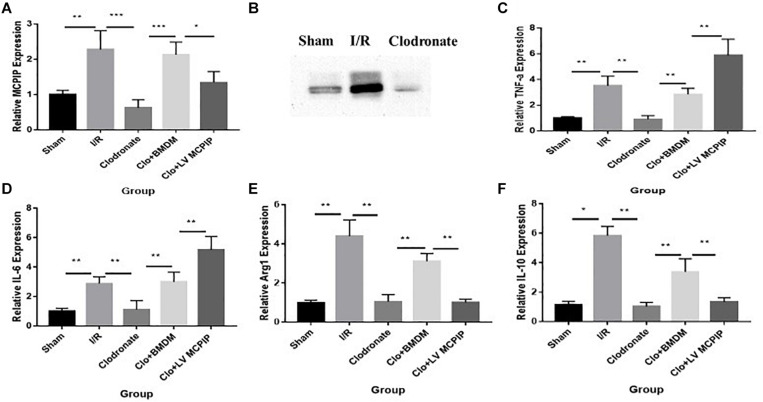
Expression levels of MCPIP and macrophage M1 and M2 markers were evaluated for mice undergoing IR injury. **(A)** Gene expression levels of MCPIP by RT-PCR in all groups. **(B)** protein expression levels of MCPIP by Western blotting analysis in the Sham, I/R, and Clodronate groups. **(C–F)** gene expression levels of M1 (TNF-a and IL-6) and M2 (IL-10 and Arg1) markers by RT-PCR in all groups. *P < 0.05, **P < 0.01, and ***P < 0.005.

We performed RT-PCR and Western blotting analysis to measure the gene and protein expression of regnase-1 in the liver (Figure 2) to ascertain its interference in this I/R injury. As demonstrated in Figure 2A, the basal MCPIP mRNA expression levels in the Sham group experienced a significant increase in the I/R group, which, however, sharply decreased in the Clodronate group with macrophage deficiency. Likewise, Western blot analysis exhibited a marked elevation of the protein level of MCPIP in the I/R group comparing with the Sham group Figure 2B, which dropped drastically in the Clodronate group. These outcomes implied that MCPIP most probably interfere in the process of liver I/R.

### To Ascertain the Localization of Regnase-1 in KCs

**FIGURE 3:**
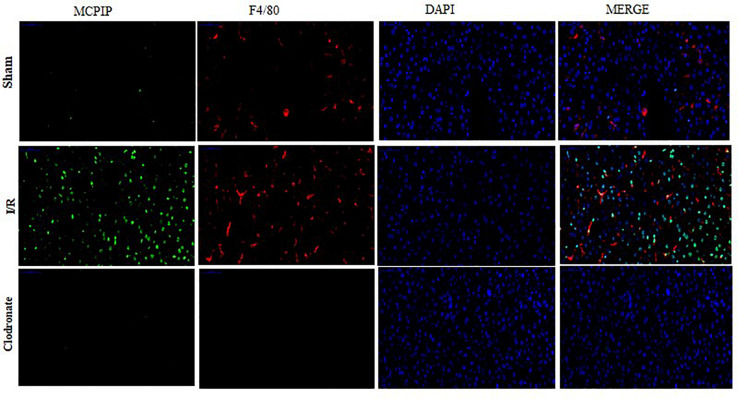
Double immunofluorescence staining was conducted to assess the expression and localization of MCPIP among Kupffer cells (KCs). MCPIP was stained green, KCs were stained with F4/80 (red), cell nuclei stained with DAPI (blue), and MCPIP (green). There were still a few luminous spots of MCPIP (green) upon macrophage exhaustion, which might support its various provenances, such as monocytes and hepatocytes.

Now that regnase-1 expression was enhanced in response to I/R but weakened in the absence of macrophages, we supposed that there might exist a relation between regnase-1 and macrophages. To test this supposition, we exploited double immunofluorescence staining to illuminate the localization and distribution of regnase-1 and KCs in liver. As illustrated in Figure 3, nuclei were counterstained with DAPI (blue), and a tiny luminescence of MCPIP (green) was scattered among the liver in the Sham group. Importantly, merging the three elements covering MCPIP (green), KCs (red), and DAPI (blue) in the Sham group was rather prominent after I/R, which, however, turned obscure in terms of macrophage exhaustion in the Clodronate group. Meanwhile, there were still a few luminous spots of MCPIP (green) upon macrophage exhaustion, which might support its various provenances, such as monocytes, and hepatocytes, in congruence with literature (Fu and Blackshear, 2017).

Taken together, our observation demonstrates right now that regnase-1 is basically kept at a relatively low level in the liver, but may become more abundant upon stimulation during I/R and is predominantly localized in KCs in agreement with previous reports.

### The Protective Role of Regnase-1 in I/R Injury

**FIGURE 4:**
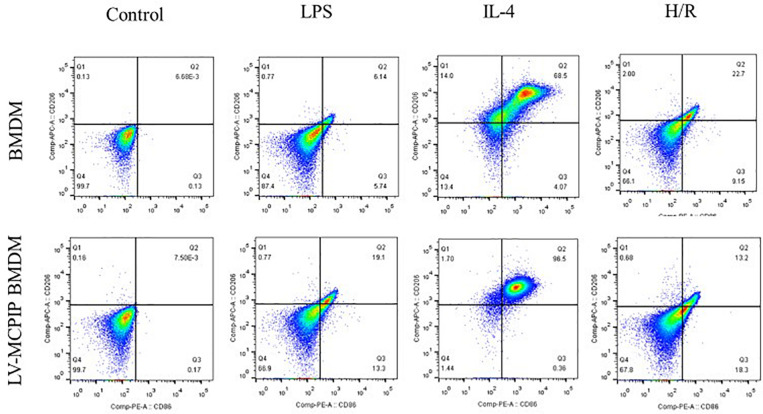
The percentage of M1 and M2 subsets of BMDM with or without MCPIP deficiency was detected by flow cytometry.

To ensure the role of regnase-1 in I/R, we adoptively transferred BMDM (M0) into mice preadministered with clodronate liposomes 1 h preoperatively to reconstitute macrophages *in vivo*. Surprisingly, even if clearance of macrophages could guard against hepatic I/R in the Clodronate group, adoptive transfer of BMDM actually resulted in deteriorated ALT and AST concentrations in the Clo + mø group (Figures 1D,E). In parallel, pathology and Suzuki’s scores also outlined pronounced impairments in this Clo + mø group (Figures 1A,C). Furthermore, this deleterious influence became even more striking in the event of transfusion of BMDM with MCPIP knockdown by LV transduction in the Clo + LV-MCPIP mø group, attached with higher levels of ALT and AST and Suzuki’s scores (Figures 1A,C–E). Of importance, data from RT-PCR demonstrates a significant difference in gene expression levels of MCPIP between BMDM and LV-MCPIP BMDM (Figure 4A), thus validating the successful knockdown efficiency by LV transduction. Thereby, results herein potentially indicate that the divergent effects between both panels of BMDM might ascribe to MCPIP competence or not. We deduce that MCPIP is capable of protecting the liver from I/R injury, whereas MCPIP knockdown abolishes this protective capability.

As it is well known that macrophages are about to release large amounts of cytokines in the context of liver I/R, a panel of cytokines were then measured to verify their potential impact on the damage in all five groups (Figure 2). By RT-PCR, gene expression levels of TNF-a and IL-6 in the WT group (Figures 2C,D), typical markers for the M1 phenotype, were significantly increased compared with the Sham group. Of importance, these increases were dramatically decreased in the absence of macrophages in the Clodronate group. Furthermore, these parameters were once more elevated in terms of macrophage reconstitution in the Clo + mø group, which, noticeably, became even more deteriorated in the case of MCPIP knockdown in the Clo + LV-MCPIP mø group. At the same time, gene expression levels of M2 markers, such as IL-10 and Arg1 (arginase 1), were investigated (Figures 2E,F), which largely displayed the opposite tendency in contrast to those of M1 markers.

Overall, based on these investigations, we have reason to believe that regnase-1 is possibly involved in liver I/R via regulation of macrophage polarization.

### To establish the Role of Regnase-1 in Macrophage M1/M2 Polarization *ex vivo*

We subsequently utilized BMDM and primary hepatocytes isolated from C57BL/6 mice to validate the impact of regnase-1 on M1/M2 states as well as the underlying mechanism *ex vivo*. Supernatants from primary hepatocytes challenged with H/R were harvested for use. BMDM (M0) were treated with 100 ng/ml LPS or 10 ng/ml IL-4 for 24 h, to induce M1 or M2 differentiation, respectively. And then, 100 μl/ml of H/R supernatants were applied to stimulate BMDM, whose polarized states were measured by flow cytometry. At the same time, LV-MCPIP BMDM with MCPIP knockdown suffered from the same protocols of stimulation; thus, we inquired into the differences between both panels of macrophages.

As illuminated by flow cytometry (Figure 4), for BMDM, LPS induced the development of the M1 type (CD11b + F4/80 + CD86 + CD206−), IL-4 stimulated M2 (CD11b + F4/80 + CD86 − CD206+), and H/R supernatants succeeded in the M1 transformation to a great extent. On the contrary, for LV-MCPIP BMDM, LPS excited an elevated frequency of M1, whereas IL-4 polarized reduced M2. It was noteworthy that, in the milieu of H/R supernatants, LV-MCPIP BMDM simultaneously posed a high percentage of M1 and low percentage of M2, and the ratio of M1/M2 was higher than that of BMDM.

**FIGURE 5:**
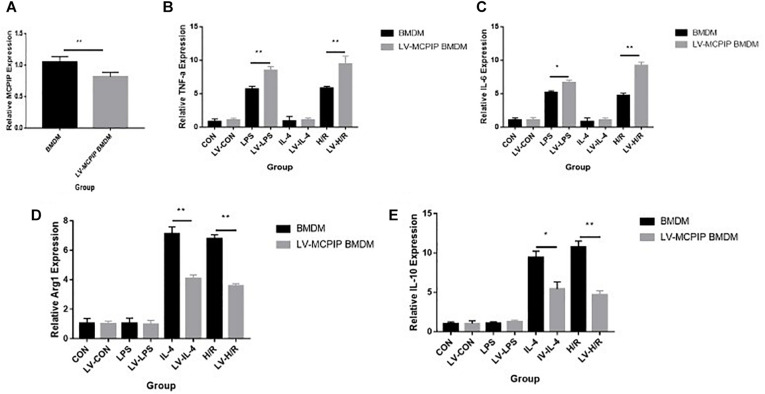
Gene expression levels of MCPIP and M1 (TNF-a and IL-6) and M2 (IL-10 and Arg1) markers in BMDM with or without MCPIP deficiency were measured by RT-PCR. *P < 0.05 and **P < 0.01. BMDM, bone marrow derived macrophage; LV-MCPIP BMDM, bone marrow derived macrophage with regnase-1 knockdown by lentiviral transduction.

Additionally, gene expressions of discriminating M1 (TNF-a and IL-6) and M2 (IL-10 and Arg1) markers were determined by RT-PCR (Figures 5B–E), and the outcomes reveal that, opposite to those for BMDM, H/R supernatants are capable of yielding enhanced M1 as well as attenuated M2 markers for LV-MCPIP BMDM.

On these bases, we could conclude now that regnase-1 does participate in the liver I/R through balancing macrophage polarizations. Although MCPIP knockdown tends to switch the M1/M2 polarization to M1, MCPIP exhibits a diverse pattern.

### The Modulatory Role of Regnase-1 in M1/M2 Transformation Through the NF-κB and CCAAT/Enhancer Binding Protein β (C/EBPβ) and Peroxisome Proliferators-Activated Receptor γ (PPARγ) Signaling Pathway

**FIGURE 6:**
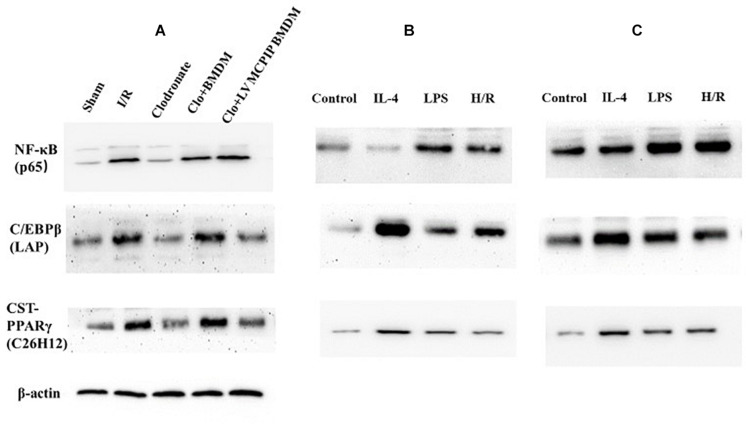
Protein levels of NF-κB p65 and C/EBPβ and PPARγ were determined by Western blotting analysis. **(A)** Representative immunoblot for these signaling proteins in mice. **(B,C)** corresponding protein levels in BMDM and BMDM with MCPIP deficiency *ex vivo*.

As NF-κB, a key player for inflammatory response, has been reported to participate in macrophage activation during liver I/R (Li X. et al., 2018), and regnase-1 has been found to suppress the NF-κB expression (Bugara et al., 2017; Ligeza et al., 2017; von Gamm et al., 2019), we next attempted to verify if regnase-1 regulated M1/M2 polarizations via NF-κB during liver I/R. As shown in Figures 6B,C, by Western blotting analysis, BMDM challenged with H/R supernatants exhibited lower levels of NF-κB p65 than LV-MCPIP BMDM subjected to the same challenges. Importantly, when both panels of macrophages encountered BAY-11-7082, a specific inhibitor for NF-κB, the differentiated phenotypes were abrogated in the presence of stimuli.

Meanwhile, NF-κB p65 in all five groups of mice were determined by Western blotting analysis (Figure 6A). The outcomes demonstrate that levels of NF-κB p65 were significantly higher in the WT group than counterparts in the Sham group (*P* < 0.05). Conversely, phosphorylations of NF-κB p65 were markedly reversed in the case of macrophage depletion in the Clodronate group, which nonetheless were strengthened once again when challenged with BMDM in the Clo + BMDM group and progressively preceded with delivery of LV-MCPIP BMDM in the Clo + LV-MCPIP BMDM group.

On the other hand, regnase-1 has been proven to facilitate M2 polarization via induction of C/EBPβ and PPARγ (Kapoor et al., 2015; Kolattukudy, 2015). We next asked whether the C/EBPβ and PPARγ signaling pathways were concerned with the transformation of M1/M2 states by regnase-1. *In vivo* (Figure 6A), Western blot detections show that C/EBPβ (LAP) expressions display a first elevated, then diminished, and then uprising, and finally impeded tendency among these five groups. Additionally, *ex vivo*, the enhancement of C/EBPβ (LAP) protein levels by H/R supernatants in BMDM was abated in LV-MCPIP BMDM (Figures 6B,C). Meanwhile, CST-PPARγ (C26H12) shared the same manifestations with C/EBPβ (LAP) by and large.

Collectively, all these results disclose that MCPIP knockdown could exacerbate the impaired lesions during liver I/R via switching M2 to M1, whose underlying mechanism is pertinent to NF-κB along with the C/EBPβ and PPARγ signaling pathways. In another word, regnase-1 could manipulate macrophage polarization through inhibition of NF-κB and facilitation of C/EBPβ and PPARγ signaling pathways.

## Discussion

Development of I/R injury, a major complication after liver resection or transplantation, contributes greatly to postoperative liver dysfunction, morbidity, and mortality. The pathophysiology of liver I/R injury not only imposes the direct ischemic insults, but also orchestrates the activation of the immunocytes and a crisscross network of their downstream inflammatory cascades. However, the mechanisms concerned are too intricate to be thoroughly interpreted thus far. At the same time, hepatic I/R retains an unresolved clinical challenge despite the countless and arduous efforts. For these reasons, we conducted the present research to explore whether the immunomodulatory properties of MCPIP were pertinent in the course of liver I/R as well as to unravel the underlying mechanisms with the hope of providing a new strategy to manage this sterile inflammation.

A successful mouse model of liver I/R was established in our research. As expected, the administration of clodronate liposomes displayed the ability to exhaust all types of macrophages *in vivo*, observed as the absence of KCs by immunohistochemistry. Opposite the observable insults in the I/R group, macrophage exhaustion in the Clodronate group enabled recovery of affected liver lesions. Later, outcomes with macrophage destruction and reconstitution conducted sequentially proved that macrophages are particularly responsible for the I/R-derived damage. In fact, numerous articles have already highlighted the specific role of macrophages in liver I/R, consistent with our findings (Yuan et al., 2016).

Macrophages are consistently accepted as potent effectors in the innate immunity, characterized by heterogeneity and plasticity (Zhou et al., 2014). On these grounds, macrophages appear to exhibit diverse properties and activation states responding to different microenvironments (Cheng et al., 2019) and to implement reversible phenotypic and functional transformation, which has been dominated as macrophage polarization.

Although activation of macrophages has been stated to initiate inflammation and regulate immunity, their contribution to the progression of liver I/R still remains contradictory, basically on account of their biphasic reactivities during this sterile inflammatory response (Li et al., 2017). As a matter of fact, macrophages have already been identified to be polarized to M1 or M2 subgroups to intervene with the I/R injury. On the one hand, in the I/R milieu, surrounding risk stimuli prompt macrophages to undergo classical differentiation into the proinflammatory M1 state so as to evoke inflammation via cytotoxic activities. On the other hand, to fight with the sustained M1 activities, clues generated by the local microenvironment alternatively allow for conversion into a special M2 status that is anti-inflammatory (Kapoor et al., 2015; Kolattukudy, 2015). More and more attention has been drawn to the role of macrophage polarization in liver I/R and how to manipulate the polarization toward a beneficial direction still puzzles clinicians.

In our study, gene and protein expression levels of MCPIP were both enhanced during hepatic I/R, which nonetheless, almost vanished in terms of macrophage exhaustion with pretreatment of clodronate liposomes. The few sporadic luminous spots labeling MCPIP left on immunofluorescence were additionally suggestive of its various cell provenances. Next to it, we found out incidentally that transfusion of genetically modified BMDM with MCPIP insufficiency engendered more exacerbated impairments than transfusion of BMDM. To dissect this divergence, we preliminarily hypothesized that MCPIP might exert a protective role in I/R, whereas MCPIP deficiency or knockdown could behave in an opposite fashion. Excited macrophages have long been recognized as a major source of a broad range of cytokines, but the different polarized states can potentiate seemingly distinct cytokine products. Subsequent detections of gene expression of relevant M1 and M2 markers by RT-PCR, in turn, reflect the intimate involvement of MCPIP in the issue of macrophage polarization.

Then, the *ex vivo* experiments firmly corroborate the regulatory role of MCPIP in macrophage polarization. When challenged by H/R supernatants, BMDM could be polarized to a certain degree of M1 and M2 states, favoring M1 by flow cytometry analysis. Amazingly, the very M1/M2 states were further directed toward M1 in the case that BMDM with MCPIP knockdown encountered the same challenges. Then, gene expression levels of M1 and M2 cytokines were additionally measured by RT-PCR, which once more supported the transformation of M2 to M1 in accord with observations by flow cytometry.

Normally, the protein regnase-1 is kept at a relatively low level. When stimulated, macrophages, the key first responders, sense stimuli and undergo a systemic intracellular signaling cascades, motivating the toll-like receptors (TLRs)/NF-κB pathway to upregulate regnase-1 expression (Mino et al., 2019). In return, regnase-1 can act to counteract unnecessary inflammatory reactions, thus composing a negative feedback loop to circumvent a disseminated cascade (Skalniak et al., 2018; Tanaka et al., 2019). Apart from its well-established role in immune modulation and defense against pathogens, regnase-1 has been held to intervene in various biological processes, such as adipogenesis, angiogenesis, cell viability, proliferation, and differentiation as well as programmed cell death (Bugara et al., 2017; Skalniak et al., 2018). MCPIP depletion or deficiency is vulnerable to diseases.

Regnase-1 has been broadly investigated in a great many diseases to exemplify its immunomodulatory capabilities in animal models, including rheumatoid arthritis, psoriasis, cardiovascular disorders, and cancer (Skalniak et al., 2018). At the same time, the modulatory role of the protein MCPIP has also been explored in some ischemic diseases. Zc3h12a−/− mice receiving middle cerebral artery occlusion manifested more pronounced attacks than WT mice (Jin et al., 2019). Whereas, in myocardial ischemia, mice with MCPIP (regnase-1) overexpression displayed ameliorated infarction and improved function via NF-κB inactivation (Jin et al., 2019).

Strenuous efforts have been made to govern the balance of M1/M2 phenotypes so as to minimize impairments inflicted by liver I/R. Regardless, whether MCPIP interferes in the progression of liver I/R and how MCPIP shapes macrophage phenotypes in this context remain unclear yet. Our current research, for the first time, uncovered the protective role of MCPIP in liver I/R, because precondition with MCPIP knockdown brought about strikingly aggravated injuries. What’s more, we ultimately affirmed that the notable protection by MCPIP was attributed to modification of macrophage polarization. MCPIP insufficiency could reprogram the M1/M2 balance toward the M1 profile, which answered to the I/R-derived stimuli by generating more pro-inflammatory cytokines as TNF-a and IL-6 and less anti-inflammatory cytokines as IL-10 and Arg1 (arginase 1).

Noticeably, few studies on the involvement of MCPIP in macrophage polarization are available to date. Kapoor et al. found that MCPIP could promote IL4-induced M2 polarization and curb M1 polarization via NF-κB inactivation in mice. This effect was further strengthened by regnase-1 overexpression and diminished by regnase-1 deletion (Kapoor et al., 2015). Our study at last confirmed the pivotal role of MCPIP in the onset of liver I/R, at least partially through modifying macrophage polarization, which is somewhat consistent with the literature. Taken together with these findings, MCPIP may potentially make a novel target to control I/R-mediated impairments in the future.

In view of the underlying mechanism, several lines of evidence have argued for the inhibitory influence of MCPIP on NF-κB (Bugara et al., 2017; Ligeza et al., 2017; von Gamm et al., 2019). The NF-κB pathway has been pointed out to take part in signaling regulation of macrophage polarization, and NF-κB inhibition can facilitate M2 activation (Kapoor et al., 2015). To see whether the NF-κB pathway was involved in our study, NF-κB p65 were detected both *in vivo* and *ex vivo*. In line with the literature, our findings ultimately uncovered that MCPIP could most probably govern macrophage polarization through suppressing NF-κB expression. In terms of MCPIP knockdown, the affected hepatic lesions challenged with I/R were further exacerbated and the balance of M1/M2 status was tipped toward M1 because NF-κB p65 was progressively sensitized.

Simultaneously, we found that MCPIP could control the M1/M2 balance by means of the C/EBPβ and PPARγ pathways and favor the M2 transformation, which is consistent with other articles (Kapoor et al., 2015; Kolattukudy, 2015). Conversely, MCPIP knockdown would likely incur M1 via suppression of C/EBPβ and PPARγ.

Overall, we are convinced right now that MCPIP is a versatile protein, and it is not only capable of inducing ROS production, ER stress, autophagy, and apoptosis in macrophages, but can also provoke macrophage polarizations in keeping with literature, chiefly due to both its deubiquitinase and RNase activities (Kapoor et al., 2015; Skalniak et al., 2018).

### Study Strengths and Limitations

There are some unavoidable drawbacks to our study. For one thing, parameters at varying time periods, such as 12, 24, and 48 h, would reflect more impressed and dynamic progression of liver I/R, which would be detected in our further study. For another, some other approaches, particularly immunohistochemistry and immunofluorescence, can also be exploited to appraise the occurrence of macrophage polarizations. Combined measurements could be more instrumental. For still another, the possibility that other important immune effector cells, such as T cells, neutrophils, NK cells, and so on, may act as confounding factors and were not completely ruled out in the present research. Additionally, macrophages with MCPIP overexpression could also be taken to authenticate the protective influence of regnase-1 during liver I/R, which will be our next work in the future. Last but not least, MCPIP deleted mice may confer more suitable and persuasive outcomes than those in our study, which nonetheless, are susceptible to immature death due to serious immune defects.

## Conclusion

In conclusion, MCPIP could play a protective role in liver I/R through regulating the macrophage M1/M2 polarization via inhibiting the NF-kB and promoting the C/EBPβ and PPARγ pathways. Upon precondition with MCPIP insufficiency, the I/R impairments are profoundly exacerbated, and the M1/M2 status was repolarized to M1 through NF-kB activation along with C/EBPβ and PPARγ suppression. Identification of the activated macrophages states and manipulation of the balance of macrophage polarization are of paramount importance and have been realized in recent years to provide important therapeutic implications for a great deal of diseases. Then, the protein MCPIP, in light of its well-established immunosuppressive properties, might serve as a promising novel target for controlling liver I/R injury, an unresolved issue clinically.

## Notes

Regnase-1, alias MCPIP1, is well known for its immunosuppressive properties and is extremely enriched in immune cells as macrophages and monocytes. Macrophage polarizations, namely M1 and M2, contribute greatly to the development of liver ischemia/reperfusion (I/R). In our current study, we first established a mouse model of liver I/R successfully *in vivo* and validated the specific role of macrophages in liver I/R. In light of increased gene and protein expression levels of regnase-1 in the I/R group and decreased levels after macrophage exhaustion in the Clodronate group, we ascertained its interference in I/R injury and its localization in macrophages. Upon macrophage reconstruction, we then found that macrophages with MCPIP deletion by lentiviral transduction caused more striking impairments compared to macrophages themselves during liver I/R. Meanwhile, gene expressions of M1 and M2 markers were examined by RT-PCR, indicating that MCPIP deletion tended to favor the M1 transformation. Subsequently, *ex vivo*, flow cytometrical detection and RT-PCR affirmed this finding. Finally, we found that MCPIP participates in macrophage M1/M2 polarization through the NF-κB, C/EBPβ, and PPARγ signaling pathways during liver I/R.

## Data Availability Statement

The raw data supporting the conclusions of this article will be made available by the authors, without undue reservation.

## Ethics Statement

The animal study was reviewed and approved by the Animal Care and Use Committee of Nanjing Medical University.

## Author Contributions

AX, JW, and CQ researched data and contributed to discussion. WJ, WB, and HC assisted in animal feeding and experiments and provided other technical assistance. AX wrote the main manuscript. KL revised the manuscript for important intellectual content. All authors read the manuscript and gave their final approval for publication.

## Conflict of Interest

The authors declare that the research was conducted in the absence of any commercial or financial relationships that could be construed as a potential conflict of interest.

## References

[B1] Behrens G. Winzen R. Rehage N. Doerrie A. Barsch M. Hoffmann A. (2018). A translational silencing function of MCPIP1/Regnase-1 specified by the target site context. *Nucleic Acids Res.* 46 4256–4270. 10.1093/nar/gky106 29471506 PMC5934641

[B2] Bugara B. Konieczny P. Wolnicka-Glubisz A. Eckhar L. Fischer H. Skalniak L. (2017). MCPIP1 contributes to the inflammatory response of UVB-treated keratinocytes. *J. Dermatol. Sci.* 87 10–18. 10.1016/j.jdermsci.2017.03.013 28377026

[B3] Cheng M.-X. Li J.-Z. Chen Y. Cao D. Gong J.-P. Tu B. (2019). VEGF-C attenuates ischemia reperfusion injury of liver graft in rats. *Transpl. Immunol.* 54 59–64. 10.1016/j.trim.2019.02.004 30771456

[B4] Cui X. Mino T. Yoshinaga M. Nakatsuka Y. Hia F. Yamasoba D. (2017). Regnase-1 and roquin nonredundantly regulate Th1 differentiation causing cardiac inflammation and fibrosis. *J. Immunol.* 199 4066–4077. 10.4049/jimmunol.1701211 29127149

[B5] Czaya B. Singh S. Yanucil C. Schramm K. Faul C. Grabner A. (2017). Induction of an inflammatory response in primary hepatocyte cultures from mice. *Jove J. Vis. Exper.* 2017:55319. 10.3791/55319 PMC5408960 28362385

[B6] Fu M. Blackshear P. J. (2017). RNA-binding proteins in immune regulation: a focus on CCCH zinc finger proteins. *Na. Rev. Immunol.* 17 130–143. 10.1038/nri.2016.129 27990022 PMC5556700

[B7] Hu Y. Yang C. Shen G. Yang S. Cheng X. Cheng F. (2019). Hyperglycemia-triggered sphingosine-1-phosphate and sphingosine-1-phosphate receptor 3 signaling worsens liver ischemia/reperfusion injury by regulating M1/M2 polarization. *Liver Transpl.* 25 1074–1090. 10.1002/lt.25470 30972941 PMC6617772

[B8] Huang H. Tohme S. Al-Khafaji A. B. Tai S. Loughran P. Chen L. (2015). Damage-associated molecular pattern-activated neutrophil extracellular trap exacerbates sterile inflammatory liver injury. *Hepatology* 62 600–614. 10.1002/hep.27841 25855125 PMC4515210

[B9] Jeltsch K. M. Hu D. Brenner S. Zoeller J. Heinz G. A. Nagel D. (2014). Cleavage of roquin and regnase-1 by the paracaspase MALT1 releases their cooperatively repressed targets to promote T(H)17 differentiation. *Nat. Immunol.* 15 1079–1089. 10.1038/ni.3008 25282160

[B10] Jin Z. Niu J. Kapoor N. Liang J. Becerra E. Kolattukudy P. E. (2019). Essential role of endothelial MCPIP in vascular integrity and post-ischemic remodeling. *Intern. J. Mol. Sci.* 20:172. 10.3390/ijms20010172 30621250 PMC6337340

[B11] Kang J.-W. Lee S.-M. (2016). Resolvin D1 protects the liver from ischemia/reperfusion injury by enhancing M2 macrophage polarization and efferocytosis. *Biochim. Biophys. Acta Mol. Cell Biol. Lipids* 1861 1025–1035. 10.1016/j.bbalip.2016.06.002 27317426

[B12] Kapoor N. Niu J. Saad Y. Kumar S. Sirakova T. Becerra E. (2015). Transcription factors STAT6 and KLF4 implement macrophage polarization via the dual catalytic powers of MCPIP. *J. Immunol.* 194 6011–6023. 10.4049/jimmunol.1402797 25934862 PMC4458412

[B13] Kolattukudy P. (2015). MCPIP: a key player in macrophage polarization. *Oncotarget* 6 28531–28532. 10.18632/oncotarget.5451 26387135 PMC4745672

[B14] Kozicky L. K. Sly L. M. (2019). Depletion and reconstitution of macrophages in mice. *Methods Mol. Biol.* 1960 101–112. 10.1007/978-1-4939-9167-9_9 30798525

[B15] Li X. Wang J. Song X. Wu H. Guo P. Jin Z. (2018). Ketamine ameliorates ischemia-reperfusion injury after liver autotransplantation by suppressing activation of Kupffer cells in rats. *Can. J. Physiol. Pharmacol.* 96 886–892. 10.1139/cjpp-2018-0046 29975111

[B16] Li Z. Zhao F. Cao Y. Zhang J. Shi P. Sun X. (2018). DHA attenuates hepatic ischemia reperfusion injury by inhibiting pyroptosis and activating PI3K/Akt pathway. *Eur. J. Pharmacol.* 835 1–10. 10.1016/j.ejphar.2018.07.054 30075219

[B17] Li Y. Ma D. Wang Z. Yang J. (2017). MicroRNA-155 deficiency in kupffer cells ameliorates liver ischemia-reperfusion injury in mice. *Transplantation* 101 1600–1608. 10.1097/tp.0000000000001765 28640790

[B18] Ligeza J. Marona P. Gach N. Lipert B. Miekus K. Wilk W. (2017). MCPIP1 contributes to clear cell renal cell carcinomas development. *Angiogenesis* 20 325–340. 10.1007/s10456-017-9540-2 28197812 PMC5511613

[B19] Mino T. Iwai N. Endo M. Inoue K. Akaki K. Hia F. (2019). Translation-dependent unwinding of stem-loops by UPF1 licenses regnase-1 to degrade inflammatory mRNAs. *Nucleic Acids Res.* 47 8838–8859. 10.1093/nar/gkz628 31329944 PMC7145602

[B20] Niu J. Wang K. Zhelyabovska O. Saad Y. Kolattukudy P. E. (2013). MCP-1-induced protein promotes endothelial-like and angiogenic properties in human bone marrow monocytic Cells. *J. Pharmacol. Exper. Therap.* 347 288–297. 10.1124/jpet.113.207316 24008336 PMC3807059

[B21] Qiu Z. Lei S. Zhao B. Wu Y. Su W. Liu M. (2017). NLRP3 inflammasome activation-mediated pyroptosis aggravates myocardial ischemia/reperfusion injury in diabetic rats. *Oxid. Med. Cell. Long.* 2017:9743280. 10.1155/2017/9743280 29062465 PMC5618779

[B22] Skalniak L. Smejda M. Cierniak A. (2018). p38 but not p53 is responsible for UVA-induced MCPIP1 expression. *Mech. Age. Dev.* 172 96–106. 10.1016/j.mad.2017.11.008 29103983

[B23] Suzuki S. Toledo-Pereyra L. H. Rodriguez F. J. Cejalvo D. (1993). Neutrophil infiltration as an important factor in liver ischemia and reperfusion injury. Modulating effects of FK506 and cyclosporine. *Transplantation* 55 1265–1272. 10.1097/00007890-199306000-00011 7685932

[B24] Takaishi M. Satoh T. Akira S. Sano S. (2018). Regnase-1, an immunomodulator, limits the IL-36/IL-36R autostimulatory loop in keratinocytes to suppress skin inflammation. *J. Invest. Dermatol.* 138 1439–1442. 10.1016/j.jid.2017.12.033 29339122

[B25] Tan L. Jiang W. Lu A. Cai H. Kong L. (2018). miR-155 aggravates liver ischemia/reperfusion injury by suppressing SOCS1 in mice. *Transpl. Proc.* 50 3831–3839. 10.1016/j.transproceed.2018.08.060 30577275

[B26] Tanaka H. Arima Y. Kamimura D. Tanaka Y. Takahashi N. Uehata T. (2019). Phosphorylation-dependent regnase-1 release from endoplasmic reticulum is critical in IL-17 response. *J. Exper. Med.* 216 1431–1449. 10.1084/jem.20181078 31072819 PMC6547859

[B27] von Gamm M. Schaub A. Jones A. N. Wolf C. Behrens G. Lichti J. (2019). Immune homeostasis and regulation of the interferon pathway require myeloid-derived Regnase-3. *J. Exper. Med.* 216 1700–1723. 10.1084/jem.20181762 31126966 PMC6605757

[B28] Wan P. Su W. Zhang Y. Li Z. Deng C. Li J. (2020). LncRNA H19 initiates microglial pyroptosis and neuronal death in retinal ischemia/reperfusion injury. *Cell Death Differ.* 27 176–191. 10.1038/s41418-019-0351-4 31127201 PMC7206022

[B29] Yang L. Wang W. Wang X. Zhao J. Xiao L. Gui W. (2019). Creg in hepatocytes ameliorates liver ischemia/reperfusion injury in a TAK1-dependent manner in mice. *Hepatology* 69 294–313. 10.1002/hep.30203 30076625

[B30] Ying W. Cheruku P. S. Bazer F. W. Safe S. H. Zhou B. (2013). Investigation of macrophage polarization using bone marrow derived macrophages. *Jove J. Vis. Exper.* 23:50323. 10.3791/50323 23851980 PMC3728835

[B31] Yu H.-C. Bai L. Yang Z.-X. Qin H.-Y. Tao K.-S. Han H. (2016). Blocking Notch signal in myeloid cells alleviates hepatic ischemia reperfusion injury by repressing the activation of NF-kappa B through CYLD. *Sci. Rep.* 6:32226. 10.1038/srep32226 27680285 PMC5041084

[B32] Yuan G. Yu Y. Ji L. Jie X. Yue L. Kang Y. (2016). Down-regulated receptor interacting protein 140 is involved in lipopolysaccharide-preconditioning-induced inactivation of Kupffer cells and attenuation of hepatic ischemia reperfusion injury. *PLoS One* 11:e0164217. 10.1371/journal.pone.0164217 27723769 PMC5056758

[B33] Zhou D. Huang C. Lin Z. Zhan S. Kong L. Fang C. (2014). Macrophage polarization and function with emphasis on the evolving roles of coordinated regulation of cellular signaling pathways. *Cell. Signal.* 26 192–197. 10.1016/j.cellsig.2013.11.004 24219909

